# ViClickbait-2025: A comprehensive dataset for Vietnamese clickbait detection

**DOI:** 10.1016/j.dib.2025.112164

**Published:** 2025-10-10

**Authors:** Dai Phuoc Nguyen, Thien Khai Tran, Y Minh Nguyen, Bay Vo

**Affiliations:** a Faculty of Information Technology, HUTECH University, Vietnam; b Faculty of Information Technology, Ho Chi Minh City University of Foreign Language – Information Technology, Vietnam; c Faculty of Information Technology, Ho Chi Minh City University of Industry and Trade, Vietnam

**Keywords:** Clickbait dataset, ViClickbait-2025, Vietnamese clickbait, Headline classification, Natural language processing, Annotation guideline, Misinformation analysis

## Abstract

ViClickbait-2025 is a curated Vietnamese-language dataset developed to facilitate research on automatic clickbait detection. It comprises 3414 headline samples collected through web scraping from eight major Vietnamese online news platforms between 2023 and 2025. Each headline is annotated as either clickbait or non-clickbait, with 31.2 % labeled as clickbait. The dataset includes nine key attributes, covering headline text, metadata, article summaries, and simulated engagement indicators. A preprocessing pipeline was applied to remove HTML noise, eliminate duplicates, and normalize the data. Annotation was carried out by three independent reviewers using a standardized guideline, with inter-annotator agreement reaching a Cohen’s Kappa of 0.822. Disagreements were resolved by a fourth annotator, and inconclusive cases were excluded. The final dataset spans 13 news categories and is released in JSONL and CSV formats under a CC BY 4.0 license.

Specifications Table

**Table:** 

Subject	Computer Sciences
Specific subject area	Natural language processing; Clickbait dataset; Vietnamese clickbait detection
Type of data	text; image
Data collection	Data were collected via automated web scraping from eight major Vietnamese news websites using Python (Requests, BeautifulSoup, Selenium). Crawling was scheduled every six hours in compliance with robots.txt. Nine fields were extracted: id, url, title, lead_paragraph, category, publish_datetime, source, thumbnail_url, and label. A two-step preprocessing pipeline was applied to clean HTML noise, remove duplicates.
Data source location	Vietnam (online sources): baomoi.com, vnexpress.net, thanhnien.vn, 24h.com.vn, vietnamnet.vn, saostar.vn, tuoitre.vn, kenh14.vn
Data accessibility	**Repository**: ViClickbait-2025: A Comprehensive Dataset for Vietnamese Clickbait Detection **Data identification number**: 10.17632/3wc46bfcjc.1 **Direct URL to data**: https://data.mendeley.com/datasets/3wc46bfcjc **Instructions for accessing these data**: For peer review only, a copy of the dataset is also mirrored at the GitHub repository below: https://github.com/blanatole/ViClickbait-2025-A-Comprehensive-Dataset-for-Vietnamese-Clickbait-Detection**(for peer review only)**
Related research article	

## 1 Value of the Data

• ViClickbait-2025 is the first openly available and curated dataset for Vietnamese clickbait detection, addressing a critical gap since most existing datasets focus on English, Turkish, Italian, or Bangla.
• The dataset comprises 3414 manually annotated headlines collected from eight diverse Vietnamese news outlets across 13 categories (*e.g.*, politics, sports, entertainment, technology), ensuring topical diversity and representativeness.
• Detailed annotation guidelines and a multi-annotator protocol (Cohen’s Kappa = 0.822) make the dataset reliable, transparent, and reusable for future research in natural language processing and media analysis.
• The dataset supports interdisciplinary studies in digital journalism, communication, and media sociology, by offering empirical evidence of headline strategies and the prevalence of clickbait practices in Vietnamese online news.

## 2 Background

Clickbait - characterized by exaggerated, emotionally charged, or misleading headlines - has become increasingly prevalent across digital media, particularly as online platforms compete for user attention. This trend raises concerns over content quality and the spread of sensationalism in digital journalism [1].

While global research on clickbait detection has progressed significantly, existing datasets are predominantly in English [2], with various resources also developed for other languages such as Turkish [3], Italian [4], and Bangla [5]. These datasets differ considerably in both size and scope. For instance, the Webis Clickbait Corpus comprises approximately 38,000 English tweets, the Turkish Clickbait Dataset contains around 48,000 Turkish headlines, and BaitBuster-Bangla includes about 253,000 annotated records. In contrast, no publicly available large-scale dataset has been developed for Vietnamese, which has constrained research progress in low-resource language settings.

Vietnamese clickbait exhibits distinctive features compared to English or Turkish, often relying on colloquial expressions, rhetorical questions, and implicit references (e.g., “người này” [this person], “điều đó” [that thing]), which reflect local journalistic styles and audience expectations. Such cultural and linguistic particularities underscore the need for a dedicated Vietnamese dataset. ViClickbait-2025 therefore represents the first openly available, curated resource for Vietnamese clickbait detection, designed to support both local NLP research and cross-lingual comparisons of clickbait strategies.

## 3 Data Description

The ViClickbait-2025 dataset consists of 3414 Vietnamese news headlines collected from eight major online media platforms between 2023 and 2025. Each record is structured with nine attributes, grouped into three categories: Metadata, Primary data, and Label (see Table 1). The Metadata group contains contextual and descriptive information, including id, url, lead_paragraph, category, publish_datetime, source, and thumbnail_url. The Main Content field captures the core textual feature: the news title. The Label indicates whether the headline is classified as clickbait or non-clickbait. Label assignment was initially guided by the editorial patterns and headline structures typical of each source, then validated through manual annotation as described in the next section.

**Table 1:** Schema and attribute description of the ViClickbait-2025 dataset.

Feature	Feature name	Data type	Definition
Primary data	title	string	Original news headline
Metadata	id	string	Unique identifier
Metadata	url	string	Link to original articl
Metadata	lead_paragraph	string	Summary of article content (if available)
Metadata	category	string	News category label
Metadata	publish_datetime	datetime	ISO 8601 formatted publication timestamp
Metadata	source	string	Name of the news website
Metadata	thumbnail_url	string	Link to article image (if available)
Label	label	string	Classification label: clickbait or non-clickbait

The dataset comprises headlines from eight Vietnamese news platforms, each contributing a different volume and proportion of clickbait content. Specifically, BaoMoi.com accounts for 731 articles (21.4 % clickbait), followed by VnExpress.net (608, 17.8 %), Thanhnien.vn (576, 16.9 %), 24h.com.vn (564, 16.5 %), Vietnamnet.vn (356, 10.4 %), Saostar.vn (286, 8.4 %), Tuoitre.vn (241, 7.1 %), and Kenh14.vn (52, 1.5 %).

**Fig. 1:**
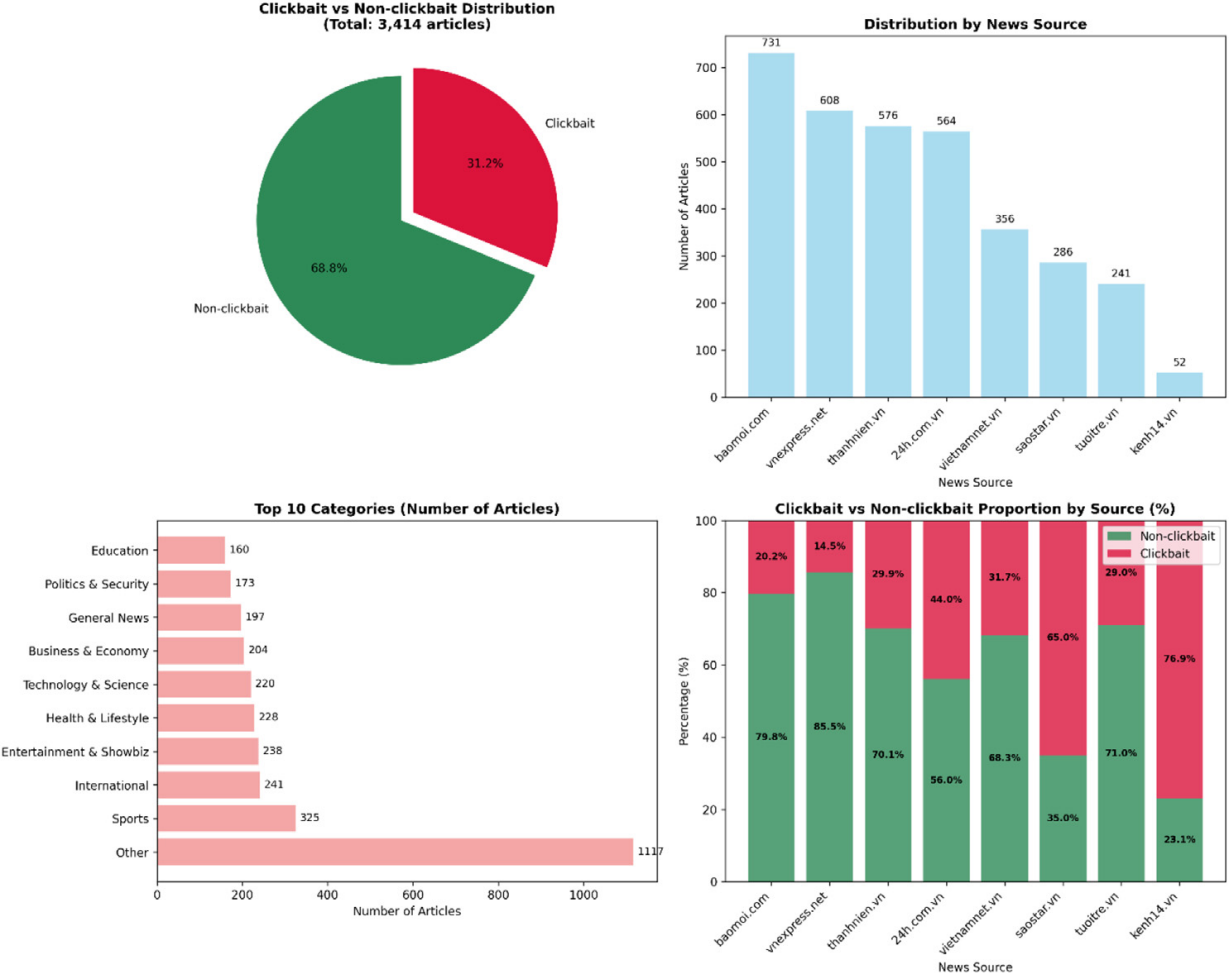
Overview of the ViClickbait-2025 dataset, comprising 3414 annotated Vietnamese news headlines across 13 categories, collected from eight online news outlets (2023–2025). Each record includes metadata, headline text, and clickbait label.

As shown in Fig. 1, the proportion of clickbait varies significantly across sources, reflecting editorial strategies and content focus. Mainstream outlets such as VnExpress and BaoMoi exhibit lower clickbait ratios, whereas entertainment-driven sites like Kenh14 and SaoStar report much higher proportions—often exceeding 65 %—suggesting a preference for emotionally charged and curiosity-driven headlines.

Analysis of headline length reveals distinct patterns between classes. Non-clickbait titles have an average length of 61.3 characters, with a narrow spread centered around the median of 60. In contrast, clickbait titles are longer and more dispersed, averaging 74.8 characters. This trend aligns with linguistic strategies commonly found in clickbait, such as elaboration, exaggeration, or ambiguity to trigger reader curiosity.

In terms of temporal coverage, the dataset is skewed toward recent content, with 2199 headlines from 2025 (64.4 %), 1154 from 2024 (33.8 %), and only 61 from 2023 (1.8 %). Despite the yearly variation in article volume, the proportion of clickbait remains relatively stable across time, ranging from 30 % to 33 %. This consistency suggests a persistent editorial practice among Vietnamese digital publishers.

**Fig. 2:**
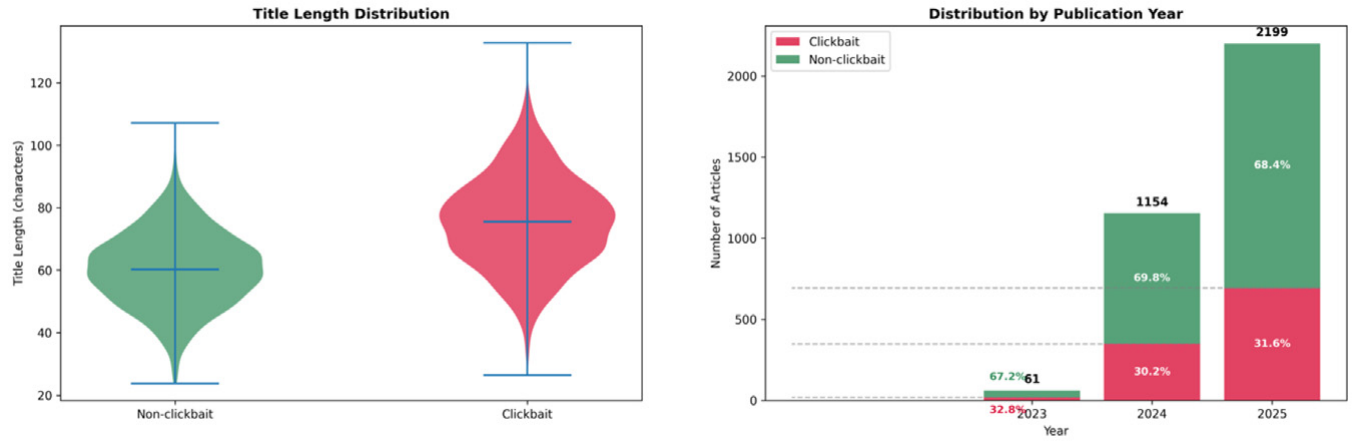
Title length and temporal distribution.

As illustrated in Fig. 2, the dataset not only reflects current publishing dynamics but also offers valuable insights into the temporal evolution of headline composition and clickbait prevalence.

## 4 Methods

**Fig. 3:**
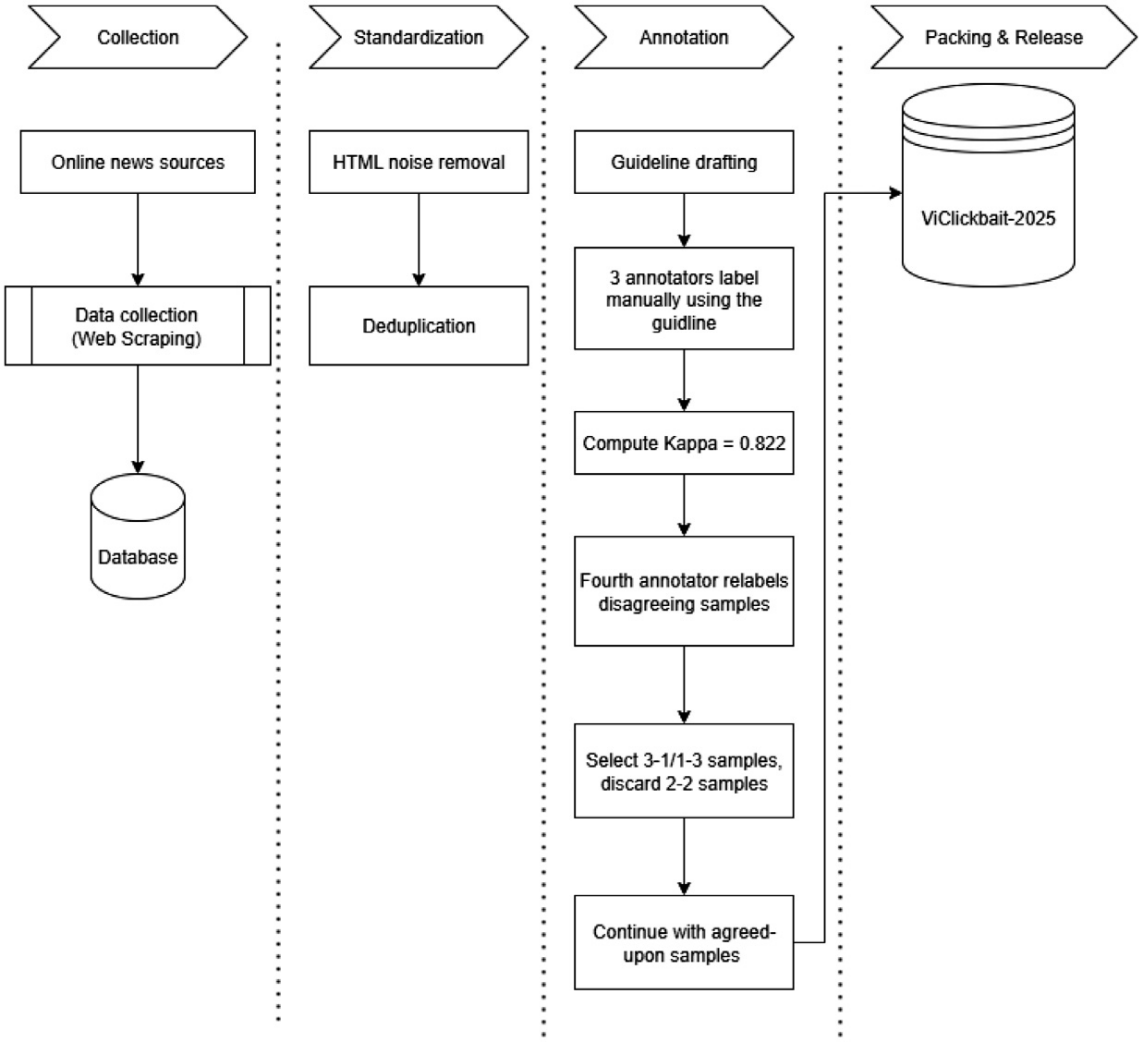
Overview of the ViClickbait-2025 dataset construction pipeline.

The creation of the ViClickbait-2025 dataset followed a systematic, five-stage pipeline designed to ensure quality, consistency, and reproducibility. The overall process included: (1) Data collection via automated web scraping from selected Vietnamese online news sources; (2) Data standardization, involving HTML cleaning and duplicate removal; (3) Manual annotation using a consensus-based protocol with inter-annotator agreement; and (4) Public release of the dataset in reusable formats with documentation and licensing. The complete construction process is illustrated in Fig. 3.

### 4.1 Collection

In the data collection phase, eight prominent Vietnamese news platforms were selected based on their popularity, topical diversity, and editorial variation. These sources include baomoi.com, vnexpress.net, thanhnien.vn, 24 h.com.vn, vietnamnet.vn, saostar.vn, tuoitre.vn, and kenh14.vn. Headlines were scraped automatically between 2023 and 2025 using Python tools such as Requests, BeautifulSoup, and Selenium, with crawling scheduled at six-hour intervals. To ensure temporal diversity, samples were gathered across various days and times. Each data point was saved with structured fields: id, url, title, lead_paragraph, category, publish_datetime, source, and thumbnail_url.

### 4.2 Standardization

In the standardization stage, all collected data underwent rigorous preprocessing. This included the removal of HTML tags, emojis, and non-standard characters using regular expressions and HTML parsing utilities. To reduce redundancy, fuzzy string matching was applied with a similarity threshold of 85 %, enabling the detection and elimination of near-duplicate records. These steps ensured both data cleanliness and sample uniqueness for reliable downstream analysis.

### 4.3 Annotation

A structured annotation protocol was established to ensure consistent and transparent labeling across the dataset. Based on prior literature and expert input, clickbait was defined as headlines intentionally crafted to attract attention and stimulate clicks by (i) creating a curiosity gap without revealing key information, (ii) using emotionally charged or exaggerated expressions, or (iii) omitting critical details. Non-clickbait headlines were defined as informative and objective, summarizing the article’s main content with sufficient contextual details (who, what, where, when) and avoiding sensational or misleading wording.

Annotators followed an eight-step guideline including quick comprehension checks, linguistic and rhetorical analysis, and comparison with the lead paragraph (when available). Specific cues were emphasized:

• Clickbait indicators: curiosity-triggering questions (“Bạn sẽ không tin…” / You won’t believe…), vague references (“người này” / this person), emotional or hyperbolic words (“gây sốc” / shocking), and listicles with unspecified content.
• Non-clickbait indicators: official statements, statistical reports, event updates, and policy announcements with concrete information.

Annotation was conducted using Label Studio, an open-source tool supporting standardized workflows and structured export formats (CSV/JSON). Three trained annotators independently labeled each headline. The three primary annotators were senior undergraduate students majoring in Information Technology/E-commerce, who received training on the annotation guideline. The fourth annotator, serving as the arbiter in disagreement cases, was a faculty member with expertise in natural language processing, ensuring methodological rigor and domain validity. Inter-annotator agreement was calculated using Cohen’s Kappa (κ), defined as:

(1) $$\kappa =\frac{Po-Pe}{1-Pe}$$

where Po represents the observed agreement and Pe the expected agreement by chance. The dataset achieved κ = 0.822, indicating substantial consistency. To resolve disagreements, a fourth annotator served as arbiter in majority-split cases (3–1 or 1–3), ensuring these items could be confidently retained. Conversely, perfect ties (2–2) were considered inherently ambiguous and excluded from the final dataset to avoid introducing noise.

### 4.4 Final packaging and release

The final dataset was released in both CSV and JSONL formats, accompanied by comprehensive metadata, usage documentation, and sample scripts to facilitate reproducibility. Licensed under Creative Commons Attribution 4.0 (CC BY 4.0), the dataset is openly accessible for research, education, and development purposes. The end-to-end pipeline emphasizes transparency and reproducibility, with the broader goal of advancing data-driven studies in Vietnamese online journalism and misinformation detection.

## Limitations

Despite its systematic construction, ViClickbait-2025 exhibits several limitations that should be considered when applying or extending the dataset.

First, the dataset is temporally imbalanced, with over 64 % of samples collected in 2025. While this ensures the dataset is up-to-date, it introduces temporal bias that may hinder model generalization across different periods, particularly if editorial styles or clickbait strategies evolve. Future iterations could incorporate historical data to improve temporal robustness.

Second, the dataset covers only eight news sources, which, although selected for diversity, do not fully capture the breadth of Vietnam’s digital media landscape. This limitation may reduce the dataset’s representativeness, particularly for regional or niche platforms with distinct writing conventions.

Third, the labeling strategy initially relied on source-based heuristics, assuming editorial consistency within each outlet. While this approach was refined through manual annotation and inter-annotator consensus, headlines from outlets with inconsistent or evolving editorial styles may still contain labeling inaccuracies. Additionally, class imbalance across categories—such as the underrepresentation of domains like “Culture & Arts” or minor sources like Kenh14.vn—could bias models toward majority classes in downstream tasks.

Fourth, although the dataset includes metadata such as thumbnail URLs, it remains strictly text-based, omitting multimodal features (e.g., images, video thumbnails) that could enhance clickbait detection in real-world applications. Integrating visual signals is a promising direction for future multimodal benchmarks.

Finally, only a subset (∼30 %) of samples received direct expert validation; the rest were accepted based on inter-annotator agreement. While this procedure yielded a high Cohen’s Kappa (0.822), borderline cases may still contain residual subjectivity or ambiguity. Further improvements could involve hierarchical labeling or tiered confidence scores to capture uncertainty in annotation.

## Ethics Statement

All procedures in this study adhered to recognized ethical standards. The research did not involve human participants, animal experimentation, or the collection of sensitive personal data. All content was obtained from publicly accessible news websites, in full compliance with each platform’s terms of service, copyright policies, and citation requirements. Data scraping was performed at responsible intervals to avoid overloading source servers. The final dataset is published under the Creative Commons Attribution 4.0 (CC BY 4.0) license, promoting transparent and ethical reuse by the broader research community.

## CRediT Author Statement

**Nguyen Phuoc Dai:** Conceptualization, Methodology, Data collection and preprocessing, Formal analysis, Original draft writing. **Thien Khai Tran:** Supervision, Method validation, Review and editing. **Nguyen Minh Y:** Data collection and preprocessing, Model experimentation, Original draft writing. **Bay Vo:** Supervision, Review and editing.

## Data Availability

Mendeley DataViClickbait-2025: A Comprehensive Dataset for Vietnamese Clickbait Detection (Original data).

## Appendix Supplementary materials

Image, application 1
